# Supplementary material for: Expression of ZNF695 Transcript Variants in Childhood B-Cell Acute Lymphoblastic Leukemia
Source: Genes (Basel). 2019 Sep 16;10(9):716. doi: 10.3390/genes10090716 (PMC6771147; doi:10.3390/genes10090716)
Supplement: Supplementary file 1 [file genes-10-00716-s001.pdf]

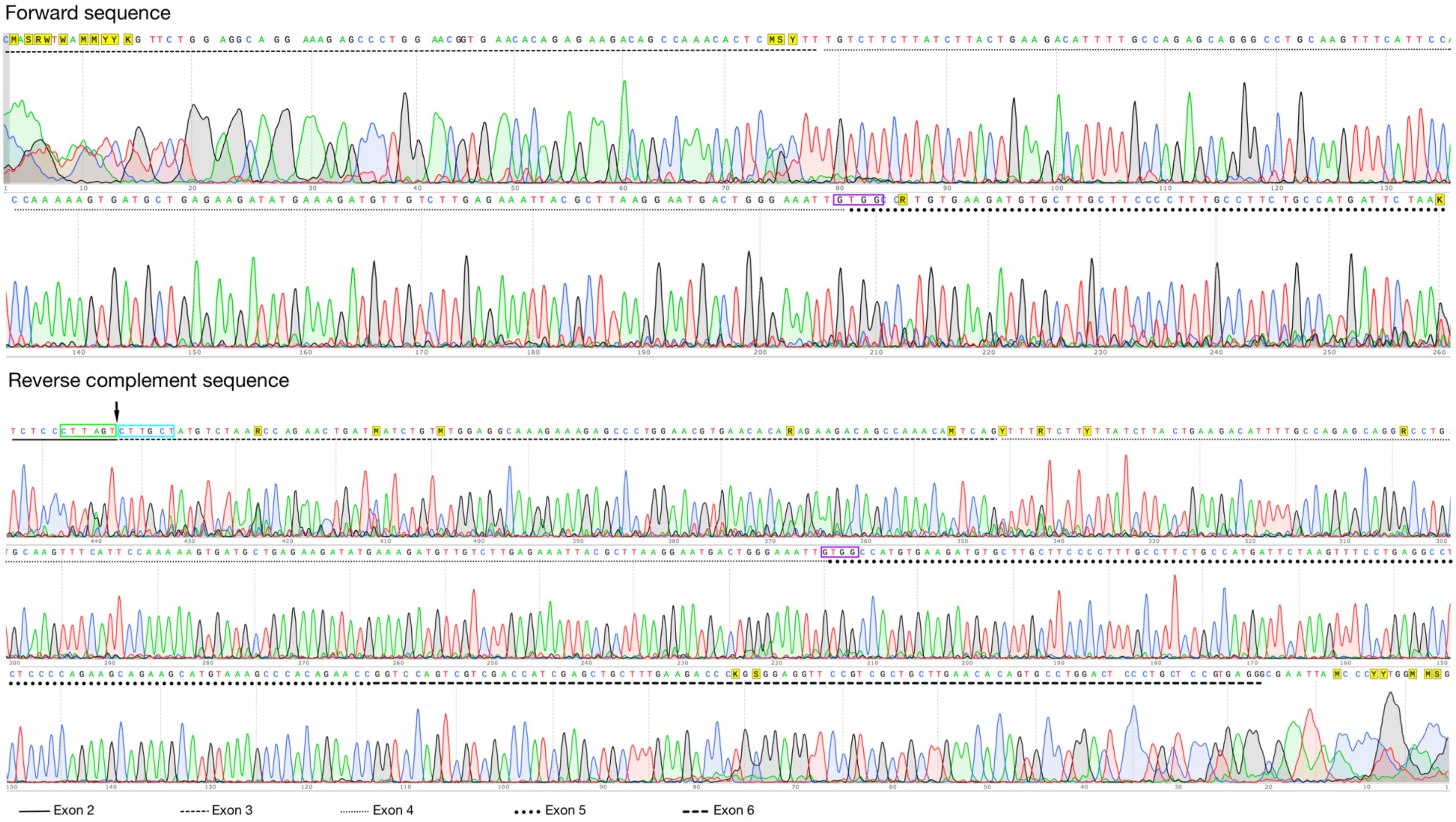

Figure S1. Representative sequence ZNF695_TV6. The electropherogram shows the forward and reverse sequences of the ZNF694 TV6 expressed in the cancer cell lines. The purple box shows the four nucleotides that identify ZNF695_TV3, the green and blue box show the boundary exon 2 and 3, respectively, and the arrow indicate the SS2. Additionally, the lines below the nucleotides indicate the sequence of each exon.

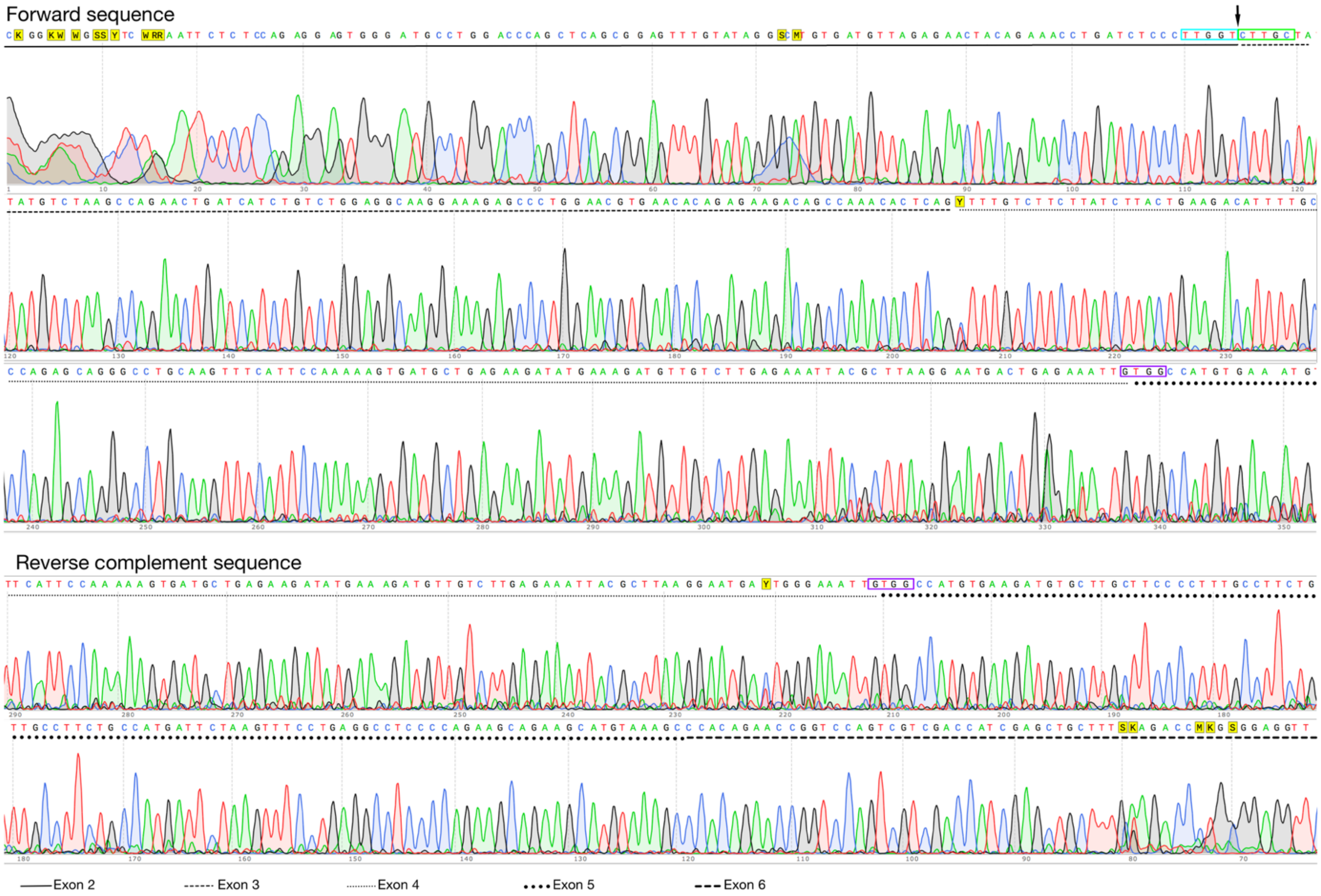

Figure S2. Representative sequence ZNF695_TV7. The electropherogram shows the forward and reverse sequences of the ZNF695 TV7 expressed in the cancer cell lines. The purple box shows the four nucleotides that identify ZNF695_TV3, the green and blue box show the boundary exon 2 and 3, respectively, and the arrow indicate the SS2. Additionally, the lines below the nucleotides indicate the sequence of each exon.
